# Neutrophil–lymphocyte ratio predicts post‐thrombolysis early neurological deterioration in acute ischemic stroke patients

**DOI:** 10.1002/brb3.1426

**Published:** 2019-09-30

**Authors:** Pengyu Gong, Yi Xie, Teng Jiang, Yukai Liu, Meng Wang, Huanhuan Sun, Shuting Zhang, Yingdong Zhang, Xiaohao Zhang, Junshan Zhou

**Affiliations:** ^1^ Department of Neurology Nanjing First Hospital Nanjing Medical University Nanjing China; ^2^ Department of Neurology Jinling Hospital Medical School of Nanjing University Nanjing China; ^3^ Department of Neurology Second Affiliated Hospital of Nanjing University of Chinese Medicine Nanjing China

**Keywords:** acute ischemic stroke, early neurological deterioration, intravenous thrombolysis, neutrophil–lymphocyte ratio, risk factor

## Abstract

**Background and purpose:**

Intravenous thrombolysis (IVT) has become the standard treatment for acute ischemic stroke within 4.5 hr after symptoms onset. However, a fraction of patients would develop early neurological deterioration (END) after IVT. The aim of our study was to explore the utility of neutrophil–lymphocyte ratio (NLR) in predicting END.

**Methods:**

From October 2016 to March 2018, 342 consecutive patients with thrombolytic therapy were prospectively enrolled in this study. Blood cell counts were sampled in stroke emergency room before IVT. END was defined as a National Institutes of Health Stroke Scale score increase of ≥4 points within 24 hr after IVT. Multiple regression analysis was used to investigate the potential risk factors of END. We also performed receiver operating characteristic curve analysis and nomogram analysis to assess the overall discriminative ability of the NLR in predicting END.

**Results:**

Of the 342 patients, 86 (25.1%) participants were identified with END. Univariate logistic regression analysis demonstrated that patients with NLR in the third tertile, compared with the first tertile, were more likely to have END (odds ratio, 9.783; 95% confidence interval [CI], 4.847–19.764; *p* = .001). The association remained significant even after controlled for potential confounders. Also, a cutoff value of 4.43 for NLR was detected in predicting post‐thrombolysis END with a sensitivity of 70.9% and a specificity of 79.3% (area under curve, 0.779; 95% CI, 0.731–0.822). Furthermore, our established nomogram indicated that higher NLR was an indicator of post‐thrombolysis END (c‐index was 0.789, *p* < .001).

**Conclusions:**

This study showed that elevated level of NLR may predict post‐thrombolysis END in ischemic stroke patients.

## Graphical abstract

This study showed that elevated level of neutrophil–lymphocyte ratio (NLR) may predict post‐thrombolysis early neurological deterioration (END) in ischemic stroke patients. A cutoff value of 4.43 for NLR was detected in predicting post‐thrombolysis END with a sensitivity of 70.9% and a specificity of 79.3% (area under curve, 0.779; 95% CI, 0.731–0.822). Furthermore, our established nomogram indicated that higher NLR was an indicator of post‐thrombolysis END (c‐index was 0.789, *p* < .001).

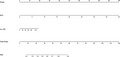

## 1 INTRODUCTION

Stroke is one of leading causes of mortality and long‐term morbidity in China (Feigin et al., 2014). Intravenous thrombolysis (IVT) with recombinant tissue plasminogen activator administered up to 4.5 hr after onset has demonstrated benefits for acute ischemic stroke (AIS) with proven efficacy in reducing mortality and long‐term morbidity (National Institute of Neurological Disorders & Stroke rt‐PA Stroke Study Group, 1995). However, within 24 hr after IVT, a fraction of patients may experience neurological deficit worsening, described as early neurological deterioration (END; Seners et al., 2017, 2014), which has been reported to be associated with poor outcomes (Mori et al., 2012). Thus, it is of great importance to explore the mechanism and the associated risk factors for post‐thrombolysis END.

Inflammation plays an important role in the pathophysiology of cerebrovascular diseases (Barone et al., 1991; Zhang et al., 2016). Except for the cytokines and chemokines released from in situ ischemic tissues, infiltration of peripheral circulating leukocytes, especially neutrophils, has been regarded as an important contributor to brain injury following ischemia (Qun et al., 2017). Several studies have confirmed the pivotal role of neutrophils in functional outcome after AIS (Gusdon et al., 2017; Wu et al., 2013). Neutrophil–lymphocyte ratio (NLR), a novel inflammatory marker that can be easily calculated from the differential white blood cell (WBC) count, was reported to be associated with the mortality and long‐term disability in stroke population (Gusdon et al., 2017; Köklü et al., 2016; Qun et al., 2017; Tokgoz et al., 2013). Also, high levels of NLR had a predictive value for 90‐day outcome of stroke patients treated with endovascular therapy (Brooks et al., 2014). However, there is a lack of data regarding the relationship between NLR and END in ischemic stroke patients underwent IVT. Therefore, we performed this prospectively observational study to explore the utility of NLR in predicting END after IVT.

## 2 METHODS

### 2.1 Study population

This prospective study was performed from October 2016 to March 2018 in Nanjing First Hospital. Patients with first‐ever AIS treated with IVT within 4.5 hr after symptom onset were included in the study. Patients treated with a bridging therapy consisting of IVT followed by endovascular therapy were also included. The exclusion criteria were as follows: (a) age <18 years and (b) unstable medical conditions such as systemic inflammatory disease, renal failure, hepatic failure, brain tumor, and presence of an active infection. Patients with hospital transfer were also excluded. Informed consent was obtained from participants or legal representatives, and the protocol was approved by the Ethical Committee of Nanjing First Hospital.

### 2.2 Clinical assessments

Clinical assessments were performed within 24 hr after admission. All participants had standard assessments of demographic characteristics, vascular risk factors (including hypertension, diabetes mellitus, dyslipidemia, current smoking, current drinking, previous stroke, atrial fibrillation, and coronary heart disease), stroke severity, stroke subtype, and laboratory data. Symptomatic intracranial hemorrhage (sICH) was defined as any hemorrhagic transformation associated with NIHSS score worsening ≥4 points. Malignant edema was considered if brain was swelling and midline shift was present together with worsening of consciousness. Computed tomography, magnetic resonance and digital subtraction angiography, and electrocardiogram, transcranial Doppler, and carotid ultrasonography were performed for assessing the stroke etiology. Stroke subtype was classified according to Trial of Org 10172 in Acute Stroke Treatment (TOAST) criteria (Adams et al., 1993). Blood cell counts, including total leukocyte, neutrophil, and lymphocyte counts, and clotting routine were sampled from each participant in stroke emergency room on admission. Then, the cell counts were analyzed by an auto‐analyzer (XE‐2100, Sysmex). NLR was calculated as neutrophil counts/lymphocyte counts.

### 2.3 Definition of END

The evaluation of neurological deficits was conducted using the National Institutes of Health Stroke Scale (NIHSS) score on admission and continued at the following 24 hr after IVT by two certified neurologists blind to clinical data. Post‐thrombolysis END was defined as a NIHSS score increase of ≥4 points between baseline and the 24 hr after IVT (Mori et al., 2012; Seners et al., 2017, 2014).

### 2.4 Treatment

All patients were treated with IVT within 4.5 hr after the onset of stroke symptoms in stroke emergency room. Once proximal arterial occlusion had been corroborated via magnetic resonance angiography or CTA, the participants would undergo rapid endovascular treatment. Other treatments, such as risk factor management and statin therapy, were also carried out as appropriate.

### 2.5 Statistical analysis

Statistical analyses were performed with SPSS version 21.0 (SPSS Inc.). Continuous variables that followed normal distribution were expressed as mean ± standard deviation; other continuous variables that did not follow normal distributions were presented as the median and the interquartile range (25th to 75th percentile). Categorical variables are expressed as constituent ratios. Differences in baseline characteristics were tested using the analysis of variance or Kruskal–Wallis test for continuous variables, and Pearson's chi‐square test for categorical variables. We also used binary logistic regression analysis to detect the risk factors of END. Multivariable analysis was adjusted for all potential confounders with statistically significant association at *p* < .1 in univariate regression analysis (including age, eGFR ≤ 60 ml/min/1.73 m^2^, initial NIHSS score, malignant edema, stroke subtype, hypersensitive C‐reactive protein, and fasting blood glucose level). Receiver operating characteristic (ROC) curve analysis was performed by assessing the overall discriminative ability of the NLR to predict post‐thrombolysis END and to establish optimal cutoff points at which the sum of the specificity and sensitivity was the highest. A MedCalc 15.6.0 (MedCalc Software) packet program was used to obtain ROC. In addition, a nomogram based on the independent predictors was constructed by R software with the package rms. The predictive capacity of the nomogram was determined by Harrell's c‐index. A two‐tailed value of *p* < .05 was considered significant.

## 3 RESULTS

From October 2016 to March 2018, 386 patients were screened in this study. Forty‐four patients were excluded for the following reasons: systemic inflammatory disease (*n* = 9), renal failure (*n* = 14), hepatic failure (*n* = 8), brain tumor (*n* = 3), and presence of an active infection (*n* = 10). A total of 342 subjects (233 men; mean age, 68.1 ± 12.3 years) were included for the final analysis. Among these patients, 238 (69.6%) had hypertension, 75 (21.9%) had diabetes mellitus, 94 (27.5%) had dyslipidemia, and 73 (21.3%) had atrial fibrillation.

After admission, END was observed in 86 patients (25.1%). The median NLR was 4.65, with tertile levels as follows: 0.66–2.27 (first tertile); 2.28–4.43 (second tertile); 4.48–35.73 (third tertile). Baseline characteristics of the study population according to the tertile of NLR are provided in Table 1. Increased NLR was related to post‐thrombolysis END (*p* = .001), onset‐to‐treatment time (*p* = .001), sICH (*p* = .006), malignant edema (*p* = .008), and high levels of fasting blood glucose (*p* = .008).

**Table 1:** Characteristics of subgroups based on the tertile of neutrophil–lymphocyte ratio

Variable	Total (*n* = 342)	First tertile (*n* = 114)	Second tertile (*n* = 114)	Third tertile (*n* = 114)	*p*
Demographic characteristics					
Age, years	68.1 ± 12.3	67.6 ± 11.4	67.9 ± 13.9	68.7 ± 11.5	.804
Male, %	233 (68.1)	80 (70.2)	76 (66.7)	77 (67.5)	.839
Vascular risk factors, %					
Hypertension	238 (69.6)	74 (64.9)	83 (72.8)	81 (71.1)	.396
Diabetes mellitus	75 (21.9)	18 (15.8)	33 (28.9)	24 (21.1)	.054
Dyslipidemia	94 (27.5)	26 (22.8)	36 (31.6)	32 (28.1)	.328
Current smoking	125 (36.5)	48 (42.1)	42 (36.8)	35 (30.7)	.202
Current drinking	94 (27.5)	33 (28.9)	30 (26.3)	31 (27.2)	.902
Previous stroke	50 (14.6)	19 (16.7)	17 (14.9)	14 (12.3)	.816
Atrial fibrillation	73 (21.3)	21 (18.4)	28 (24.6)	24 (21.1)	.525
Coronary heart disease	65 (19.0)	16 (14.0)	22 (19.3)	27 (23.7)	.188
Clinical data					
Previous antiplatelet, %	60 (17.5)	26 (22.8)	21 (18.4)	13 (11.4)	.074
Previous statin, %	22 (6.4)	11 (9.6)	8 (7.0)	3 (2.6)	.086
eGFR ≤ 60 ml/min/1.73 m^2^, %	97 (28.4)	25 (21.9)	34 (29.8)	38 (33.3)	.147
Systolic blood pressure, mmHg	147.3 ± 22.3	147.8 ± 25.2	148.6 ± 20.8	145.5 ± 20.6	.549
Diastolic blood pressure, mmHg	87.4 ± 14.4	89.0 ± 16.3	86.5 ± 13.1	86.9 ± 13.6	.371
Body mass index, kg/m^2^	23.9 ± 3.4	24.1 ± 3.1	24.2 ± 3.4	23.6 ± 3.7	.330
Initial NIHSS, score	8 (3, 13)	8 (3, 12)	6 (3, 12)	9 (4, 15)	.067
OTT, min	149.0 (104.0, 200.0)	120.0 (96.0, 170.0)	158.0 (105.0, 210.0)	180.5 (115.0, 215.0)	.001
Proximal arterial occlusion, %	105 (32.4)	34 (29.8)	30 (26.3)	44 (38.6)	.121
Endovascular treatment, %	82 (25.3)	23 (20.2)	24 (21.1)	35 (30.7)	.118
Post‐thrombolysis END, %	86 (25.1)	12 (10.5)	13 (11.4)	61 (53.5)	.001
sICH, %	23 (6.7)	2 (1.8)	7 (6.1)	14 (12.3)	.006
Malignant edema, %	16 (4.7)	3 (2.6)	2 (0.6)	11 (9.6)	.008
Stroke subtype, %					.504
LAA	98 (28.7)	38 (33.3)	28 (24.6)	32 (28.1)	
CE	91 (26.6)	30 (26.3)	35 (30.7)	26 (22.8)	
SAO	77 (22.5)	26 (22.8)	26 (22.8)	25 (21.9)	
Others or undetermined	76 (22.2)	20 (17.5)	25 (21.9)	31 (27.2)	
Laboratory data					
TC, mmol/L	4.4 ± 1.1	4.6 ± 1.1	4.4 ± 1.1	4.4 ± 1.1	.278
TG, mmol/L	1.3 (0.9, 1.7)	1.3 (0.9, 2.1)	1.3 (0.9, 1.7)	1.2 (0.8, 1.6)	.081
LDL, mmol/L	2.7 (2.0, 3.3)	2.8 (2.1, 3.6)	2.7 (2.0, 3.4)	2.6 (1.9, 3.2)	.160
Hs‐CRP, μg/ml	4.6 (2.0, 8.6)	3.6 (2.0, 6.7)	4.7 (1.9, 8.4)	6.1 (2.0, 9.8)	.087
FBG, mmol/L	6.2 ± 2.0	5.8 ± 1.8	6.2 ± 2.2	6.6 ± 1.8	.008
Homocysteine, μmol/L	16.3 ± 8.2	16.8 ± 6.9	15.3 ± 7.6	16.6 ± 10.0	.358
Uric acid, μmol/L	308.3 ± 109.2	319.3 ± 108.9	312.6 ± 107.7	293.1 ± 110.2	.172

Abbreviations: CE, cardioembolism; eGFR, estimate glomerular filtration rate; FBG, fasting blood glucose; Hs‐CRP, hypersensitive c‐reactive protein; LAA, large artery atherosclerosis; LDL, low‐density lipoprotein; NIHSS, National Institutes of Health Stroke Scale; OTT, onset‐to‐treatment time; SAO, small artery occlusion; sICH, symptomatic intracranial hemorrhage; TC, total cholesterol; TG, triglyceride.

Comparisons of baseline characteristics in patients with or without END are shown in Table 2. Compared with patients without post‐thrombolysis END, patients with post‐thrombolysis END were older (*p* = .016) and had higher proportions of proximal arterial occlusion (*p* = .001); lower proportions of previous antiplatelet (*p* = .020); lower levels of body mass index (*p* = .028) and triglyceride (*p* = .015); higher levels of initial NIHSS (*p* = .001), hypersensitive C‐reactive protein (*p* = .001), fasting blood glucose (*p* = .001), and NLR (*p* = .001).

**Table 2:** Characteristics of subgroups based on the presence of post‐thrombolysis early neurological deterioration

Variable	END group (*n* = 86)	Non‐END group (*n* = 256)	*p*
Demographic characteristics			
Age, years	70.9 ± 11.4	67.2 ± 12.7	.016
Male, %	58 (67.4)	158 (61.7)	.874
Vascular risk factors, %			
Hypertension	63 (73.3)	175 (68.4)	.393
Diabetes mellitus	13 (15.1)	62 (24.2)	.078
Dyslipidemia	25 (29.1)	69 (27.0)	.704
Current smoking	29 (33.7)	96 (37.5)	.529
Current drinking	30 (34.9)	64 (25.0)	.076
Previous stroke	7 (8.1)	43 (16.8)	.100
Atrial fibrillation	21 (24.4)	52 (20.3)	.421
Coronary heart disease	21 (24.4)	44 (17.2)	.144
Clinical data			
Previous antiplatelet, %	8 (9.3)	52 (20.3)	.020
Previous statin, %	3 (3.5)	19 (7.4)	.181
eGFR ≤ 60 ml/min/1.73 m^2^, %	31 (36.0)	66 (25.8)	.068
SBP, mmHg	145.4 ± 19.5	148.0 ± 23.1	.360
DBP, mmHg	86.7 ± 12.8	87.7 ± 14.9	.563
Body mass index, kg/m^2^	23.3 ± 3.3	24.2 ± 3.4	.028
Initial NIHSS, score	12 (6, 18)	7 (3, 11)	.001
OTT, min	175.0 (110.0, 205.0)	141.5 (100.0, 200.0)	.068
Proximal arterial occlusion, %	53 (61.6)	85 (33.2)	.001
Stroke subtype, %			.073
LAA	47 (54.7)	111 (43.4)	
CE	21 (24.4)	69 (27.0)	
SAO	6 (7.0)	45 (17.6)	
Others or undetermined	12 (14.0)	31 (12.1)	
Laboratory data			
TC, mmol/L	4.4 ± 1.0	4.4 ± 1.1	.986
TG, mmol/L	1.1 (0.8, 1.5)	1.2 (0.9, 1.9)	.015
LDL, mmol/L	2.6 (2.0, 3.2)	2.7 (2.0, 3.4)	.567
Hs‐CRP, μg/ml	6.5 (2.8, 11.6)	3.7 (1.8, 7.5)	.001
FBG, mmol/L	6.9 ± 1.8	5.9 ± 1.9	.001
Homocysteine, μmol/L	17.2 ± 9.3	16.0 ± 7.8	.254
Uric acid, μmol/L	309.8 ± 111.9	307.8 ± 108.5	.886
NLR	6.8 (2.6, 11.8)	2.6 (1.7, 4.1)	.001

Abbreviations: CE, cardioembolism; DBP, diastolic blood pressure; eGFR, estimate glomerular filtration rate; FBG, fasting blood glucose; Hs‐CRP, hypersensitive c‐reactive protein; LAA, large artery atherosclerosis; LDL, low‐density lipoprotein; NIHSS, National Institutes of Health Stroke Scale; OTT, onset‐to‐treatment time; SAO, small artery occlusion; SBP, systolic blood pressure; TC, total cholesterol; TG, triglyceride.

Table 3 showed the results of logistic regression analysis for risk factors with post‐thrombolysis END. Univariate logistic regression analysis demonstrated that the highest tertile of NLR, age, previous antiplatelet, eGFR ≤ 60 ml/min/1.73 m^2^, initial NIHSS score, BMI, proximal arterial occlusion, sICH, malignant edema, hypersensitive C‐reactive protein, and fasting blood glucose level were associated with END. After adjusting for all potential confounders, the highest tertile of NLR (first quartile used as the reference value) was identified as an independent predictor for post‐thrombolysis END (odds ratio [OR], 6.406; 95% confidence interval [CI] 2.646–15.510, *p* = .002).

**Table 3:** Logistic regression analysis for risk factors with post‐thrombolysis early neurological deterioration

Variable	OR	95% CI	*p*
Crude model			
Demographic characteristics			
Age, years	1.021	1.001–1.043	.046
Male	0.959	0.569–1.616	.874
Vascular risk factors			
Hypertension	1.268	0.735–2.187	.394
Diabetes mellitus	0.622	0.328–1.180	.146
Dyslipidemia	1.029	0.596–1.775	.919
Current smoking	0.848	0.507–1.417	.529
Current drinking	1.495	0.881–2.536	.136
Previous stroke	0.494	0.211–1.160	.105
Atrial fibrillation	1.267	0.711–2.260	.422
CHD	1.549	0.859–2.794	.146
Clinical data			
Previous antiplatelet	0.402	0.183–0.885	.024
Previous statin	0.438	0.126–1.517	.193
eGFR ≤ 60 ml/min/1.73 m^2^	1.623	0.963–2.734	.069
SBP, mmHg	0.995	0.984–1.006	.359
DBP, mmHg	0.995	0.978–1.012	.562
Body mass index, kg/m^2^	0.920	0.853–0.992	.029
Initial NIHSS, score	1.083	1.044–1.123	.001
OTT time, min	1.001	0.997–1.005	.570
Proximal arterial occlusion	3.852	2.307–6.433	.001
sICH	6.549	2.669–16.072	.001
Malignant edema	2.432	0.877–6.742	.088
TOAST subtype, %			
LAA	1.572	0.767–3.220	.217
CE	1.240	0.588–2.613	.572
Other and undetermined	1.794	0.850–3.785	.125
SAO	Reference	Reference	
Laboratory data			
TC, mmol/L	1.002	0.798–1.258	.986
TG, mmol/L	0.842	0.628–1.128	.248
LDL, mmol/L	0.999	0.989–1.008	.770
Hs‐CRP, μg/ml	1.050	1.021–1.080	.001
FBG, mmol/L	1.246	1.103–1.408	.001
Homocysteine, μmol/L	1.017	0.988–1.048	.256
Uric acid, μmol/L	1.000	0.998–1.002	.886
NLR distribution			
First tertile	Reference	Reference	
Second tertile	1.094	0.476–2.513	.832
Third tertile	9.783	4.847–19.764	.001
Adjusted model^a^			
NLR distribution			
First tertile	Reference	Reference	
Second tertile	1.101	0.401–3.025	.852
Third tertile	6.406	2.646–15.510	.002

Abbreviations: CE, cardioembolism; CHD, coronary heart disease; CI, confidence interval; DBP, diastolic blood pressure; FBG, fasting blood glucose; GFR, estimate glomerular filtration rate; Hs‐CRP, hypersensitive c‐reactive protein; LAA, large artery atherosclerosis; LDL, low‐density lipoprotein; NIHSS, National Institutes of Health Stroke Scale; NLR, neutrophil–lymphocyte ratio; OR, odds ratio; OTT, onset‐to‐treatment time; SAO, small artery occlusion; SBP, systolic blood pressure; sICH, symptomatic intracranial hemorrhage; TC, total cholesterol; TG, triglyceride.

a Adjusted model was controlled for age, previous antiplatelet, eGFR ≤ 60 ml/min/1.73 m^2^, initial NIHSS score, body mass index, proximal arterial occlusion, sICH, malignant edema, hypersensitive C‐reactive protein, and fasting blood glucose level.

Of particular interest, IVT patients were further divided into those with and without endovascular therapy. Compared with the first tertile, NLR in the third tertile showed a higher trend for the occurrence of post‐thrombolysis END (Table 4; OR, 7.064; 95% CI, 3.009–16.583; *p* = .001) in patients without endovascular therapy. Furthermore, in bridging therapy group, higher NLR level remained associated with post‐thrombolysis END.

**Table 4:** Subgroup analysis according to the patients undergoing intravenous thrombolysis with and without endovascular therapy

NLR distribution	IVT patients with endovascular therapy (*n* = 82)			IVT patients without endovascular therapy (*n* = 260)		
	OR	95% CI	*p*	OR	95% CI	*p*
First tertile	Reference	Reference		Reference	Reference	
Second tertile	0.950	0.207–4.350	.947	1.153	0.424–3.135	.781
Third tertile	22.958	5.712–92.279	.001	7.064	3.009–16.583	.001

Abbreviations: CI, confidence interval; IVT, intravenous thrombolysis; NLR, neutrophil–lymphocyte ratio; OR, odds ratio.

**Figure 1:**
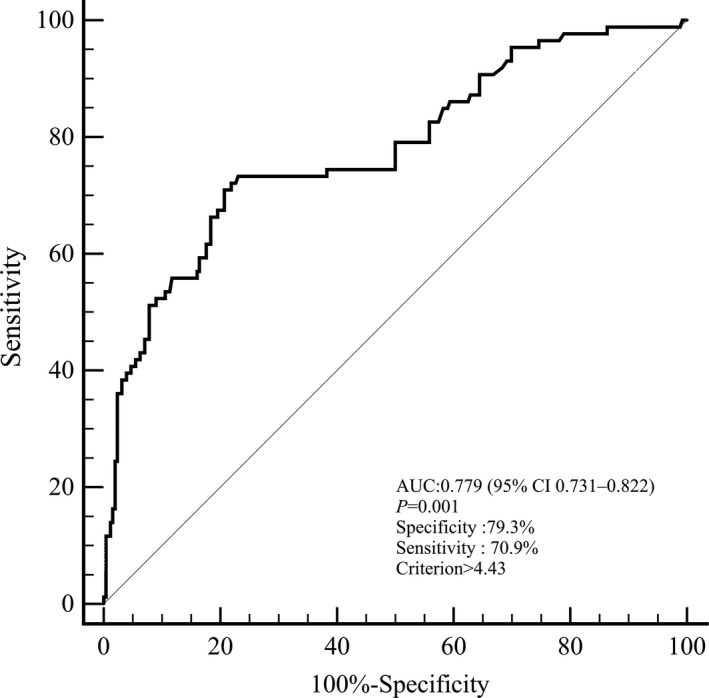
Receiver operating characteristic (ROC) curve for the value of neutrophil–lymphocyte ratio (NLR) to predict post‐thrombolysis early neurological deterioration (END)

As for the clinical value of NLR in predicting post‐thrombolysis END, we performed ROC curve in Figure 1. The optimal cutoff value for NLR as a predictor of post‐thrombolysis END was determined as 4.43 in the ROC curve analysis, which yielded a sensitivity of 70.9% and a specificity of 79.3%, with the AUC at 0.779 (95% CI, 0.731–0.822).

**Figure 2:**
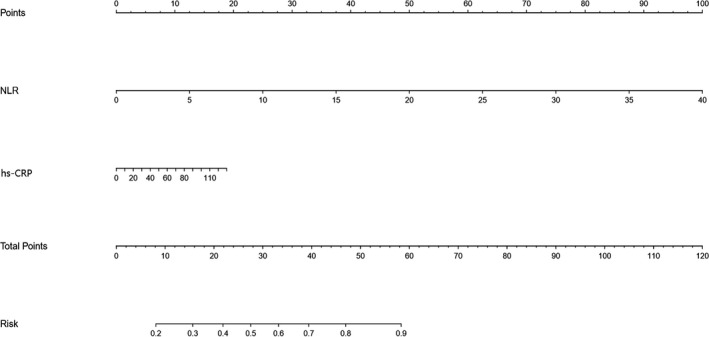
Nomograms of acute ischemic stroke patients to predict post‐thrombolysis early neurological deterioration (END)

The nomogram is shown in Figure 2. The concordance index of this model was 0.789 (*p* < .001). The novel models indicated that higher NLR was an indicator of post‐thrombolysis END. These findings were similar to those obtained previously in the multivariate logistic models.

We divided the patients into group A (NLR < 4.43) and group B (NLR ≥ 4.43). Comparisons of characteristics based on the two groups are shown in Table 5. Compared with group B, patients in group A had less time from onset to treatment (*p* = .003), higher proportions of previous statin (*p* = .034) and proximal arterial occlusion (*p* = .001), higher levels of triglyceride (*p* = .034), lower levels of hypersensitive C‐reactive protein (*p* = .033), and fasting blood glucose (*p* = .007).

**Table 5:** Characteristics of group A and group B

Variable	Group A (*n* = 227)	Group B (*n* = 115)	*p*
Demographic characteristics			
Age, years	67.8 ± 12.8	68.8 ± 11.7	.502
Male, %	155 (68.2)	78 (67.8)	.932
Vascular risk factors, %			
Hypertension	156 (68.7)	82 (71.3)	.624
Diabetes mellitus	50 (22.0)	25 (21.7)	.952
Dyslipidemia	62 (27.3)	32 (27.8)	.920
Current smoking	90 (39.6)	35 (30.4)	.095
Current drinking	62 (27.3)	32 (27.8)	.920
Previous stroke	36 (15.9)	14 (12.2)	.489
Atrial fibrillation	48 (21.1)	25 (21.1)	.899
Coronary heart disease	38 (16.7)	27 (23.5)	.139
Clinical data			
Previous antiplatelet, %	46 (21.6)	14 (12.2)	.063
Previous statin, %	19 (8.4)	3 (2.6)	.034
eGFR ≤ 60 ml/min/1.73 m^2^, %	59 (26.0)	38 (33.0)	.172
SBP, mmHg	148.3 ± 23.1	145.4 ± 20.5	.264
DBP, mmHg	87.7 ± 14.8	86.9 ± 13.6	.607
Body mass index, kg/m^2^	24.2 ± 3.3	23.6 ± 3.6	.154
Initial NIHSS, score	7 (3, 12)	9 (4, 15)	.052
OTT, min	130.0 (100.5, 192.5)	183.0 (116.5, 215.0)	.003
Proximal arterial occlusion, %	77 (33.9)	61 (53.0)	.001
Stroke subtype, %			.076
LAA	98 (43.2)	60 (52.2)	
CE	64 (28.2)	26 (22.6)	
SAO	40 (17.6)	11 (9.6)	
Others or undetermined	25 (11.0)	18 (15.7)	
Laboratory data			
TC, mmol/L	4.5 ± 1.8	4.4 ± 1.1	.537
TG, mmol/L	1.3 (0.9, 1.9)	1.1 (0.8, 1.5)	.001
LDL, mmol/L	2.7 (2.1, 3.5)	2.6 (1.9, 3.2)	.128
Hs‐CRP, μg/ml	4.0 (1.9, 7.2)	6.1 (2.0, 9.8)	.033
FBG, mmol/L	6.0 ± 2.0	6.6 ± 1.8	.007
Homocysteine, μmol/L	16.1 ± 7.2	16.5 ± 10.0	.712
Uric acid, μmol/L	315.6 ± 108.3	293.9 ± 110.0	.081

Abbreviations: CE, cardioembolism; DBP, diastolic blood pressure; eGFR, estimated glomerular filtration rate; FBG, fasting blood glucose; Hs‐CRP, hypersensitive c‐reactive protein; LAA, large artery atherosclerosis; LDL, low‐density lipoprotein; NIHSS, National Institutes of Health Stroke Scale; OTT, onset‐to‐treatment time; SAO, small artery occlusion; SBP, systolic blood pressure; TC, total cholesterol; TG, triglyceride.

## 4 DISCUSSION

In this study, we unveiled that NLR, an affordable and readily available tool, may be a powerful predictor of END in AIS patients being considered for IVT. Our study showed that patients with elevated NLR levels were at increasing risks of developing post‐thrombolysis END, even when controlling for age, proximal arterial occlusion, sICH, malignant edema, and other potential confounders. Furthermore, the optimal cutoff value of NLR to indicate post‐thrombolysis END was 4.43, and its corresponding sensitivity and specificity were 70.9% and 79.3%, respectively. Furthermore, our established nomogram indicated that higher NLR was an indicator of post‐thrombolysis END.

Clinical evidences have shown that, in AIS patients, high NLR levels are associated with increased infarct volume and mortality (Celikbilek, Ismailogullari, & Zararsiz, 2014; Gökhan et al., 2013; Tokgoz et al., 2013). Furthermore, NLR is a predictor of recurrent ischemic stroke and 90‐day poor functional outcome in AIS patients receiving endovascular stroke therapy or IVT or antiplatelet medications (Brooks et al., 2014; Duan et al., 2018; Malhotra et al., 2018; Qun et al., 2017). In the present study, it is the first time to investigate the relationship between NLR and early functional deterioration in AIS patients with IVT therapy. In accordance with previous studies (Rajajee et al., 2006; Seners, Turc, Oppenheim, & Baron, 2015; Thanvi, Treadwell, & Robinson, 2008), our cohort reported a prevalence of 25.1% in post‐thrombolysis END. We found that NLR revealed its predictive value in the occurrence of post‐thrombolysis END. It is worthwhile to note that, in bridging therapy group, NLR still has great potential as a predictor of END occurrence, suggesting the types of treatment may have minimal effects on the relationship between NLR and END following AIS.

Mechanistically, END is believed to be resulted from biochemical abnormality such as inflammation (Alawneh, Moustafa, & Baron, 2009; Zhang et al., 2016). END in patients with lacunar infarction has reported a correlation with high peripheral concentrations of pro‐inflammatory factors, such as IL‐6, TNF‐α, and intercellular adhesion molecule‐1 (Castellanos et al., 2002). In AIS, it is reported that the inflammatory process is launched within 24 hr at ischemic site and has an important role in exacerbating ischemic damage (Kim, Park, Chang, Kim, & Lee, 2016; Zhang, Chopp, Chen, & Garcia, 1994). The inflammatory cytokines and chemokines released from ischemic tissues guide the infiltration of circulating leukocytes, among which neutrophils are the most recognized mediator in ischemic brain injury (Wu et al., 2013). Neutrophil, the main inflammatory cell of AIS, on one hand, can release free oxygen radicals propagating secondary brain injury in penumbra regions (Ceulemans et al., 2010). On the other hand, neutrophils are the source of matrix metalloproteinase‐9 (MMP‐9), which can directly result in blood–brain barrier (BBB) breakdown and hemorrhagic transformation (Duan et al., 2018). These may be significant contributors in the occurrence of END. Additionally, some subtypes of lymphocyte have been reported to be major cerebroprotective immunomodulators after AIS in response to ischemic injury and are involved in reduced infarct volume and improved neurological function (Kim et al., 2012; Liesz et al., 2013). Therefore, higher lymphocyte count may be related to lower risks of END. However, single biomarker is prone to be affected by various physiological and pathological conditions (Nash et al., 2014). NLR, a composite parameter in the combination of neutrophils and lymphocytes, could serve to better reflect immunological activities of the cells and divide patients into comprehensive inflammatory profiles, playing a better role in predicting post‐thrombolysis END in patients with AIS.

Our study has some potential limitations. Firstly, our study was conducted within participants from one single center via strict exclusion criteria, whose results might not be able to generalize to the general population. Secondly, the sample size of the present study was relatively small. Larger cohorts of subjects are needed. Thirdly, it has been proposed that blood cell counts may change during the recovery of ischemic stroke (Iadecola & Anrather, 2011). To maximally reduce the possible correlation of this effect with our results, blood cell counts were assessed before IVT to minimize the time interval between the onset of stroke and blood sampling. Fourthly, although we found the relationship between NLR and post‐thrombolysis END, there did not exist dynamic examination of the blood cell count of every patient. Blood cell count needed to be examined dynamically in further studies. Finally, data were observational. We were therefore unable to establish a causal relationship between NLR and END after IVT.

In conclusion, from the present study, NLR levels appeared to be positively correlated with post‐thrombolysis END in ischemic stroke patients and can serve as a useful noninvasive biomarker for assessment of END after IVT.

## CONFLICT OF INTEREST

All the authors declare that there is no conflict of interest.

## AUTHOR CONTRIBUTIONS

All authors listed have contributed significantly and are in agreement with the content of the manuscript. Pengyu Gong was mainly involved in study design, data analysis, data acquisition, data interpretation, and manuscript preparation. Xiaohao Zhang was mainly involved in study design, data analysis, data interpretation, and manuscript preparation. Teng Jiang was mainly involved in data analysis, data interpretation, and manuscript preparation. Yi Xie was mainly involved in data interpretation and manuscript preparation. Yukai Liu was mainly involved in data acquisition and data analysis. Meng Wang, Huanhuan Sun, and Shuting Zhang were mainly involved in data acquisition. Junshan Zhou and Yingdong Zhang were mainly involved in study design, data interpretation, and manuscript preparation.

## Data Availability

The data that support the findings of this study are available from the corresponding author upon reasonable request.
